# Association Between the Introduction of Pediatric Influenza Vaccination and Influenza Diagnoses in Primary Care and Hospitalizations: An Interrupted Time Series Study

**DOI:** 10.3390/vaccines14050372

**Published:** 2026-04-22

**Authors:** Sílvia Burgaya-Subirana, Anna Ruiz-Comellas, Queralt Miró-Catalina, Judit Dorca Vila, Núria Rovira Girabal, Montse Ruiz, Mónica Balaguer

**Affiliations:** 1 Pediatrics Department, EAP Manlleu, Institut Català de la Salut, Central Catalonia Primary and Community Care Management, C/Castellot, 17, 08560 Manlleu, Spain; sburgaya.cc.ics@gencat.cat; 2 Faculty of Medicine, University of Vic-Central University of Catalonia (UVIC-UCC), 08500 Vic, Spain; monica.balaguer@umedicina.cat; 3 Family Medicine Department, EAP Sant Joan de Vilatorrada, Institut Català de la Salut, Central Catalonia Primary and Community Care Management, Avinguda del Torrent del Canigó 0, 08250 Sant Joan de Vilatorrada, Spain; 4 Research Group on Health Promotion in Rural Areas, Jordi Gol i Gurina Primary Care Research Institute, 08242 Manresa, Spain; 5 Research Support Unit, Institut Català de la Salut, Central Catalonia Primary and Community Care Management, C/Pica d’Estats 13-15, 08272 Sant Fruitós de Bages, Spain; qmiro.cc.ics@gencat.cat; 6 Pediatrics Department, Althaia, University Healthcare Network of Manresa, C/Dr. Joan Soler 1–3, 08243 Manresa, Spain; 7 Pediatrics Department, Berga Hospital, Salut Catalunya Central, Cta/de Ribes s/n, 08600 Berga, Spain; 8 Pediatrics Department, Vic University Hospital, C/Pla el Vigatà 1, 08500 Vic, Spain; 9 Pediatric Intensive Care Unit, Sant Joan de Déu Hospital, Passeig de Sant Joan de Déu 2, 08950 Esplugues de Llobregat, Spain

**Keywords:** infections, hospitalizations, influenza, pediatrics, influenza vaccine, systeatic vaccination

## Abstract

Introduction: Influenza has a major impact on public health. The best way to prevent it is through vaccination. In Catalonia, influenza vaccination has been recommended for children aged 6 to 59 months since the 2023–24 season. Objective: To assess the association between the implementation of this vaccination program and changes in influenza diagnoses in primary care and influenza-related hospitalizations in all age groups. Materials and Methods: Quasi-experimental study with interrupted time series (ITS) analysis. All influenza diagnoses made in primary care (PC) and all influenza-related hospitalizations in the Central Catalonia health region between October 2018 and August 2025 were included. The monthly aggregated cases were analyzed using segmented negative binomial regression models that accounted for temporal trends, the onset of COVID-19, and the introduction of systematic pediatric influenza vaccination. Results: A total of 6804 influenza diagnoses made in PC and 3252 hospitalizations for influenza were analyzed. A statistically significant decrease was observed in the percentage of influenza diagnoses in PC in the 2–4 (13.5% vs. 10.6%) and 5–14 (26.1% vs. 16.3%) age groups. In the ITS analysis conducted in primary care (PC) settings, the vaccination period was significantly associated with a 13% reduction in expected influenza cases among individuals aged 15–64 years (RR 0.87 [0.78; 0.99]). After sensitivity analysis, these results were no longer statistically significant. The ITS analysis in the hospital setting has not shown a significant reduction in expected influenza cases or in expected admissions. Conclusions: Systematic influenza vaccination in children aged 6 to 59 months has not been shown to be associated with a reduction in influenza cases in primary care or hospitals settings during the early stages of implementation of the new vaccination program.

## 1. Introduction

Seasonal influenza is an infectious respiratory disease caused by the influenzae virus (a virus from the Orthomyxoviridae family). There are four subtypes, A, B, C, and D, but only subtypes A and B cause clinically relevant disease in humans and are responsible for epidemics [1].

The World Health Organization estimates that approximately one billion infections occur annually, with between 3 and 5 million severe cases and up to 650,000 deaths [2].

The annual incidence of influenza in children is higher than in adults and older adults [3,4,5,6]; nevertheless, individuals aged >65 years usually receive greater attention during each epidemic because mortality in this group is higher than in the other age groups [1,3,4].

Therefore, children under 5 years of age are the most susceptible to influenza infection and are also the most vulnerable to severe disease [4,7]. In addition, the pediatric population has been identified as the most efficient vector for the spread of influenza in the community, since children shed higher viral loads in respiratory secretions and for a longer period of time than adults [1,4,8].

Vaccination is the most effective strategy to prevent influenza and its complications [8]. Influenza vaccination in pediatric populations is considered one of the most effective preventive strategies. It provides direct protection by reducing hospitalizations and severe disease, as well as indirect protection by decreasing transmission to others, particularly high-risk groups of all ages and older adults [1,8,9,10,11].

Although universal influenza vaccination is currently carried out in healthy children in more than a third of countries worldwide, its rollout throughout history has been uneven. The pioneers in introducing universal influenza vaccination at pediatric age were the United States and Canada during the 2003–04 season. However, there are still differences between countries regarding the age groups included in routine vaccination and the types of influenza vaccines used [11].

In Southern Europe, including Spain, the rollout of universal pediatric influenza vaccination has been relatively recent and variable across regions.

In Spain, influenza vaccination in children was traditionally recommended only for those aged over 6 months with underlying risk conditions [Table A1] [12]. However, following national and international recommendations, universal influenza vaccination for children aged 6 to 59 months was introduced in several regions during the 2023/24 season, including Catalonia [13]. This recent policy change provides a valuable opportunity to assess the impact of universal pediatric vaccination in a real-world Southern European context.

The aim of this study is to assess the association between the introduction of systematic influenza vaccination in children aged 6 to 59 months and changes in the number of influenza diagnoses in primary care (PC) and hospitalizations for influenza for all age groups in the Central Catalonia health region, Spain.

## 2. Materials and Methods

### 2.1. Study Design and Participants

This is a quasi-experimental study with interrupted time series analysis conducted in the Central Catalonia health region. This is a region located in northeastern Spain that provides healthcare coverage to a population of 400,000 people. This region has 39 PC centers and three reference hospitals (University Hospital of Vic, Althaia. Manresa Healthcare Network and Berga Hospital, Vic, Spain). This study included all patients of any age clinically diagnosed with influenza who were seen in PC between 1 October 2018 and 31 August 2025. Likewise, all influenza admissions to any of the three hospitals in this region during the same time period were included.

Influenza records in primary care (PC) refer to children with a clinical diagnosis of influenza before December 2020, as rapid influenza tests were not available in PC prior to that date. After December 2020, patients included in this study were those diagnosed with influenza, whether clinical or confirmed by a rapid test. Patients diagnosed with influenza in hospital settings had either a rapid diagnostic test or a PCR test for the influenza virus.

Primary care data were obtained from the electronic database of the Institut Català de la Salut (ICS), the main public health provider in Catalonia, and hospitalization data were obtained from the electronic database of each of the hospitals in the region.

### 2.2. Variables

The variables collected for each patient diagnosed with influenza at the PC centers were as follows: age, sex, influenza severity (mild/severe), underlying condition considered a risk factor for complications after an influenza virus infection (yes/no), and prior influenza vaccination in any of the seasons studied. Mild or severe influenza was defined based on the codes recorded in the primary are database. The codes that were considered severe were those that associated influenza with pneumonia, encephalopathy and myocarditis (J09.X1, J10.00, J11.00, J11.08, J10.01, J10.08, J11.81, J11.82, J11.83, J10.81, J10.82, J10.83). The codes considered mild were those that did not associate influenza with any other condition (J11.1, J11.2, J10.1, J10.2, J09.X2, J09.X9, J09.X3, J10.89, J11.89). The underlying conditions considered to be at risk that were taken into account in children were those recorded in Table A1, and in adults those recorded in Table A2. For patients admitted to each of the hospitals, the following variables were collected: age, sex, and days of hospitalization. The pre-vaccination period was considered to be those seasons prior to the introduction of systematic influenza vaccination in children under 5 years of age (2018/19–2022/23), and the post-vaccination period was considered to be those seasons after the introduction of the new vaccination program (2023/24 and 2024/25).

### 2.3. Statistical Analysis

Influenza cases or admissions were described according to the reported sociodemographic variables: sex, age, severity, and risk in primary care, and sex, age, and length of stay for hospitals. The description was carried out for the overall total, depending on whether the person was vaccinated or not, and also according to the case period: pre- vaccination and post-vaccination. The start of vaccination was considered to be 1 October 2023. The description was carried out using percentages, absolute counts, and the chi-square (χ2) test. The aggregated cases by month are shown in charts. The graphs were produced for the general population and also by age group to identify possible differences in the impact of vaccination. An interrupted time series analysis was conducted using monthly aggregated data and negative binomial regression models. The model includes two immediate changes associated with the onset of COVID-19 (March 2020) and the introduction of influenza vaccination in children under 5 years of age (October 2023) and a change in the subsequent slope for each of the two events. A single model was estimated for PC and another for hospitals, controlling for age, and then a model was fitted for each age group. To control for the large volume of contrasts for each group, Bonferroni correction was applied to the *p*-values and confidence intervals of the estimated RR for each model. A sensitivity analysis was also performed to assess the robustness of the model’s results. All analyses were carried out using R version 4.2.1, and the significance level was set at 5%.

### 2.4. Ethical Considerations

This research was conducted in accordance with the Declaration of Helsinki and Spanish national and institutional legislation concerning clinical research and the protection of personal data. The data collected from the databases of both the ICS and each of the hospitals participating in the study were pseudoanonymized by the technical department of each organization, so that no member of the research team could identify the participants. For this reason, informed consent was not required.

This study was approved by the ethics committee of the IDIAP Jordi Gol (Code: 23/139; Approval date: 26 July 2023) and by the IRIS-CC Ethics Committee (Code: 23/001; Approval date: 13 October 2023).

## 3. Results

### 3.1. Influenza Diagnoses in Primary Care

In total, 6804 influenza diagnoses in PC were studied throughout the entire study period. The majority (87%) of the reported cases had not been vaccinated during the study period. Most vaccinated cases corresponded to adults (26.9% in the 15–64 age group and 51.5% in those over 65 years) [Table A3]. A total of 76.8% had no underlying conditions.

The post-vaccination period was associated with a significant reduction in the percentage of influenza diagnoses in the 2–4 (13.5% vs. 10.6%) and 5–14 age groups (26.1% vs. 16.3%), as well as a reduction in diagnoses of complicated influenza (1.91% vs. 0.88%) [Table 1].

**Figure 1:**
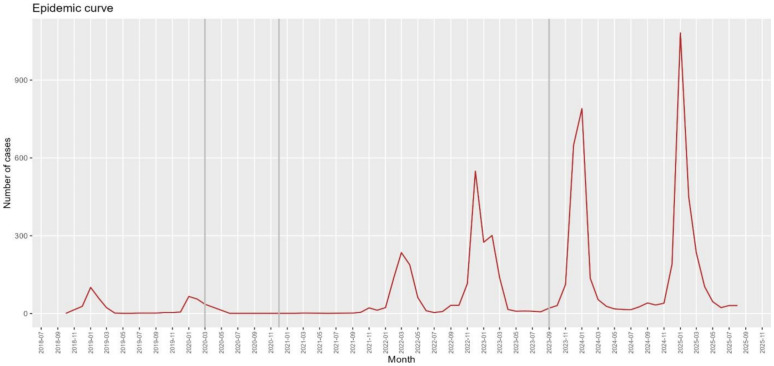
Seasonal trend in the number of influenza cases in PC. The first vertical gray line corresponds to the start of the COVID-19 pandemic, the second corresponds to the introduction of rapid diagnostic tests in PC, and the third to the start of systematic influenza vaccination in children under 5 years of age.

In Figure 1 we observe that during the 2020–21 season, practically no influenza diagnoses were observed (only four were recorded in the whole of Central Catalonia), and these increased in a generalized manner in subsequent seasons.

### 3.2. Influenza Hospitalizations

**Figure 2:**
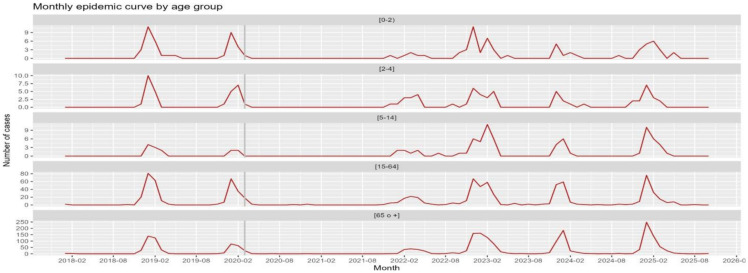
Evolution by season and by age group of hospitalizations for influenza. The vertical gray line corresponds to the start of the COVID-19 pandemic. Impact of influenza vaccination in children under 5 years of age.

During the study period, there were 3252 hospitalizations for influenza. The majority (64%) were in those aged 65 years and over. Regarding the pediatric age (<15 years), the age group with the most hospitalizations was 0–2 years. In children under 5 years of age, a reduction in the percentage of admissions was associated with the post-vaccination period (4.12% vs. 2.34% in the 0–2 group and 3.43% vs. 2.06% in the 2–4 age group). This decrease could also be observed in the 15 to 64 age group (32.8% to 23.2%). In any case, these associations were not statistically significant in any of the groups mentioned. No statistically significant differences were observed either in terms of days of hospitalization between the two periods [Table 2 and Figure 2].

In Figure 2 we see that during the 2020–21 season (COVID-19 pandemic), virtually no influenza-related hospitalizations were observed. It can also be observed that during the 2023/24 and 2024/25 seasons, influenza hospital admissions decreased in the 0–2, 2–4, and 15–64 age groups compared with the seasons prior to the COVID-19 pandemic.

To assess the impact of influenza vaccination in children under 5 years of age, an interrupted time series (ITS) analysis was conducted both at the primary care level and at the hospital level.

In PC settings, the vaccination period was significantly associated with a 13% reduction in expected influenza cases among individuals aged 15–64 years (RR 0.87 [0.78; 0.99]) [see Supplementary Table S2]. No statistically significant association was observed, although RRs across the other age groups were below 1 [see Supplementary Tables S1–S3].

Regarding hospitalizations, no statistically significant association was observed either at the start or throughout the vaccination period [see Supplementary Tables S1–S3]. After applying a sensitivity analysis excluding the 2020/21 season (COVID-19 season), none of the results remained statistically significant [see Supplementary Tables S4–S6].

## 4. Discussion

This study assesses the association between the introduction of systematic influenza vaccination in children aged 6 to 59 months in Catalonia and trends in influenza diagnoses in PC and influenza-related hospitalizations across all age groups. The results provide limited evidence of an association between the introduction of the new vaccination program and a reduction in influenza cases in primary care and hospital settings. This pattern is consistent with the early implementation phase of a vaccination program, when coverage remains limited and the follow-up period is relatively short.

Children play a central role in the community transmission of influenza, as they have a higher viral load and longer shedding periods than adults. For this reason, pediatric vaccination can provide both direct protection in vaccinated children and indirect effects in other age groups [1,4,8,10,11]. Experiences in European countries with established childhood influenza vaccination programs have shown substantial population-level im- pacts [14]. For example, in the United Kingdom, the gradual introduction of influenza vaccination in school-aged children was associated with significant reductions in consultations for influenza-like illness, emergency department visits, and hospitalizations in both pediatric and adult populations [4]. Similarly, European systematic reviews have described that childhood vaccination programs can generate measurable indirect effects in adults and older adults when high vaccination coverage is achieved [15,16,17,18]. In our study, a significant association with a decrease in the percentage of influenza diagnoses in PC was observed only in the 2–4 and 5–14 age groups after the introduction of the vaccination program. In the ITS analysis, the vaccination period was significantly associated with a 13% reduction in expected influenza cases among individuals aged 15–64 years; however, after applying a sensitivity analysis excluding the 2020/21 season (COVID-19 season), these results were no longer statistically significant. Moreover, no clear reduction was detected in hospitalizations compared with pre-pandemic levels, which likely reflects the interaction of several epidemiological factors. First, the introduction of rapid diagnostic tests for influenza in PC starting in December 2020 likely reduced the underdiagnosis present in previous seasons and increased case detection [19]. Before the availability of these tests, the diagnosis of influenza was based primarily on clinical criteria, with limited sensitivity, which contributed to underdiagnosis and the classification of many cases as nonspecific respiratory infections [20]. Rapid tests, although they have moderate sensitivity (approximately 50–70%), show high specificity (>95%), allowing confirmation of a greater number of true cases [21]. At the population level, their implementation may lead to an apparent increase in recorded incidence due to the reduction of prior underdiagnosis.

Second, vaccination coverage in children during the program’s first seasons was modest, with approximate values of 19% and 27% in the 2023/24 and 2024/25 seasons [22]. These coverage levels are lower than those described in established pediatric vaccination programs and may be insufficient to generate a clear population-level indirect effect, as several epidemiological models suggest that coverage above 50–60% is necessary to produce a substantial community impact [15,16,17,18]. The United Kingdom’s experience during the 2014–15 season, with an average coverage of 56% among children aged 4 to 11, resulted in a 94% reduction in primary care consultations for influenza-like illness, a 74% decrease in emergency visits for respiratory disease, and a 93% reduction in hospitalizations for laboratory-confirmed influenza among schoolchildren. A community benefit was also observed, with a 59% decrease in consultations for influenza-like illness in adults [4]. The annual variability in the effectiveness of the influenza vaccine may also have influenced the observed results, given its dependence on the degree of antigenic match between the vaccine strains and circulating viruses. In Spain, during the 2023–2024 season, influenza A (H1N1)pdm09 predominated, with most circulating viruses belonging to clade 5a.2a, whereas the vaccine strain corresponded to subclade 5a.2a.11, suggesting a close but not complete antigenic match. This partial concordance may have contributed to the relatively favorable vaccine effectiveness observed, while still allowing for some attenuation of its impact. In contrast, the 2024–2025 season was characterized by the co-circulation of influenza A and B viruses, indicating greater viral heterogeneity. Although this pattern is broadly compatible with the composition of quadrivalent vaccines, intra-subtype variability, particularly for A (H3N2), may still have limited overall vaccine effectiveness. These seasonal differences in strain match may therefore have contributed to variability in the estimated impact of the vaccination program across seasons [23,24]. Among this study’s limitations, it is worth highlighting the potential detection biases resulting from changes in diagnostic strategies and the impact of the COVID-19 pandemic on the circulation of respiratory viruses, which makes comparability between periods more difficult. In addition, the vaccination coverage observed during the first seasons of the program was relatively low, which could limit the emergence of indirect effects at the population level. Furthermore, the ecological nature of this analysis introduces the possibility of ecological fallacy, as associations observed at the population level cannot be directly extrapolated to the individual level to infer vaccine effectiveness. Therefore, the findings should be interpreted with caution, and future studies based on individual-level data will be necessary to validate these results and to better estimate the direct and indirect effects of vaccination. However, this study has relevant strengths, such as the use of real-world data from both primary care and hospital settings, as well as the application of an interrupted time-series design to analyze the impact of a public health intervention at the population scale. Future studies with longer follow-up periods will be required to confirm the population-level impact of pediatric influenza vaccination in contexts with progressively increasing coverage. Likewise, analysis of data at the national or multi-center level could allow for a more precise estimation of the program’s indirect effects on vulnerable age groups, especially in the population over 65 years of age. 

## 5. Conclusions

The introduction of systematic influenza vaccination in children aged 6 to 59 months has not been associated with a reduction in influenza cases in primary care or hospital settings during the early stages of the new vaccination program’s implementation. Longer follow-up and higher vaccination coverage are required to confirm its population-level impact and maximize its potential benefits.

Higher vaccination coverage is needed to confirm its population-level impact and maximize the potential benefits of vaccination.

## Figures and Tables

**Table 1:** Description of cases diagnosed with influenza in PC. Vaccination period.

	Total Cases	Pre-Vaccination	Pos-Vaccination	
			*p*-Value	
	n (%)	n (%)	n (%)	
Total cases	6804	2623	4181	
Sex			0.479	
Male	3251 (47.8%)	1268 (48.3%)	1983 (47.4%)	
Female	3553 (52.2%)	1355 (51.7%)	2198 (52.6%)	
Age				<0.001
[0–2)	448 (6.58%)	180 (6.86%)	268 (6.41%)	0.495
[2–4)	800 (11.8%)	355 (13.5%)	445 (10.6%)	<0.001
[5–14)	1366 (20.1%)	685 (26.1%)	681 (16.3%)	<0.001
[15–64)	3310 (48.6%)	1143 (43.6%)	2167 (51.8%)	<0.001
[65 or over)	880 (12.9%)	260 (9.91%)	620 (14.8%)	<0.001
Severity				<0.001
Mild	6717 (98.7%)	2573 (98.1%)	4144 (99.1%)	
Complicated	87 (1.28%)	50 (1.91%)	37 (0.88%)	
Risk				0.007
Yes	1580 (23.2%)	563 (21.5%)	1017 (24.3%)	
No	5224 (76.8%)	2060 (78.5%)	3164 (75.7%)	

**Table 2:** Description of hospitalizations for study period.

	Total Cases	Pre-Vaccination	Post-Vaccination	*p*-Value
	n (%)	n (%)	n (%)	
Total cases	3252	1794	1458	
Sex				0.368
Male	1563 (48.1%)	714 (49.0%)	849 (47.3%)	
Female	1689 (51.9%)	744 (51.0%)	945 (52.7%)	
Age				<0.001
[0–2)	102 (3.14%)	60 (4.12%)	42 (2.34%)	0.513
[2–4)	87 (2.68%)	50 (3.43%)	37 (2.06%)	0.742
[5–14)	84 (2.59%)	29 (1.99%)	55 (3.07%)	<0.001
[15–64)	896 (27.6%)	478 (32.8%)	418 (23.2%)	0.212
[65 or over)	2079 (64.0%)	839 (57.6%)	1240 (69.2%)	<0.001
Length of stay	6.51 (8.73)	6.51 (8.73)	6.40 (9.25)	0.425

## Data Availability

The data that support the findings of this study are available from the corresponding author upon request.

## Supplementary Materials

The following supporting information can be downloaded at: https://www.mdpi.com/article/10.3390/vaccines14050372/s1, Table S1. Adjusted negative binomial regressions for changes over time and by age groups. 0–2 years group and 2–4 years group. Vaccination period time: change in trend during the vaccination period. Vaccination start period: im- 374 mediate effect of the vaccination period. Time since COVID onset: change in trend after the onset of COVID. COVID onset: immediate effect of the onset of COVID. Time: temporal trend. Table S2. Adjusted negative binomial regressions for changes over time and by age groups. 5–14 years group and 15–64 years group. Vaccination period time: change in trend during the vaccination period. Vaccination start period: im- 374 mediate effect of the vaccination period. Time since COVID onset: change in trend after the onset of COVID. COVID onset: immediate effect of the onset of COVID. Time: temporal trend. Table S3. Adjusted negative binomial regressions for changes over time and by age groups. >65 years group. Vaccination period time: change in trend during the vaccination period. Vaccination start period: im- 374 mediate effect of the vaccination period. Time since COVID onset: change in trend after the onset of COVID. COVID onset: immediate effect of the onset of COVID. Time: temporal trend. Table S4. Adjusted negative binomial regressions for changes over time and by age groups after applying a sensitivity test. (The 2020/21 season has been excluded). 0–2 years group and 2–4 years group. Vaccination period time: change in trend during the vaccination period. Vaccination start period: im- 374 mediate effect of the vaccination period. Time since COVID onset: change in trend after the onset of COVID. COVID onset: immediate effect of the onset of COVID. Time: temporal trend. Table S5. Adjusted negative binomial regressions for changes over time and by age groups after applying a sensitivity test. (The 2020/21 season has been excluded). 5–14 years group and 15–64 years group. Vaccination period time: change in trend during the vaccination period. Vaccination start period: im- 374 mediate effect of the vaccination period. Time since COVID onset: change in trend after the onset of COVID. COVID onset: immediate effect of the onset of COVID. Time: temporal trend. Table S6. Adjusted negative binomial regressions for changes over time and by age groups after applying a sensitivity test. (The 2020/21 season has been excluded). >65 years group. Vaccination period time: change in trend during the vaccination period. Vaccination start period: im- 374 mediate effect of the vaccination period. Time since COVID onset: change in trend after the onset of COVID. COVID onset: immediate effect of the onset of COVID. Time: temporal trend.

## Abbreviations

The following abbreviations are used in this manuscript:

PC	Primary care
ICS	Institut Català de la Salut
ITS	Interrupted time series

## Appendix A

**Table A1:** Risk factors that the Department of Health of Catalonia has considered as indications for receiving the influenza vaccine in children older than 6 months through the 2022/23 season.

Risk Factors for Influenza Vaccination in Children
- Chronic cardiovascular, neurological, or respiratory diseases (including hypertension, asthma, bronchopulmonary dysplasia, and cystic fibrosis).
- Diabetes mellitus.
- Morbid obesity: body mass index (BMI) in adolescents ≥ 35 and ≥3 standard deviations in children.
- Chronic kidney disease and nephrotic syndrome.
- Hemoglobinopathies and anemia.
- Hemophilia and other coagulation disorders, chronic bleeding disorders, recipients of blood products, and multiple transfusions.
- Asplenia or severe asplenic dysfunction.
- Chronic liver disease.
- Severe neuromuscular diseases.
- Immunosuppression (including primary immunodeficiencies and those caused by HIV infection, drugs, including treatment with eculizumab, or in transplant recipients) and complement deficiencies.
- Cancer and malignant blood disorders.
- Cochlear implant or awaiting an implant.
- Cerebrospinal fluid fistula.
- Celiac disease.
- Chronic inflammatory disease.
- Disorders and diseases that entail cognitive dysfunction: Down syndrome, etc.
- Children and adolescents receiving prolonged treatment with acetylsalicylic acid, because of the possibility of developing Reye syndrome after influenza.
- Children institutionalized for an extended period.
- Children between 6 months and 2 years old with a history of prematurity of less than 32 weeks gestation.

**Table A2:** Underlying conditions that the Department of Health of the Government of Catalonia considers to be risk factors for developing complications after an influenza infection in adults. People with these conditions are candidates to receive the influenza vaccination [12,13].

Underlying Conditions Considered to Be a Risk for Complications After an Influenza Infection in Adults
Chronic cardiovascular, neurological, or respiratory diseases (including hypertension, asthma, bronchopulmonary dysplasia, and cystic fibrosis).
- Diabetes mellitus.
- Morbid obesity: body mass index (BMI) ≥ 40 in adults and ≥35 in adolescents.
- Chronic kidney disease and nephrotic syndrome.
- Hemoglobinopathies and anemia.
Hemophilia and other coagulation disorders, chronic bleeding disorders, recipients of blood products, and multiple transfusions.
- Asplenia or severe asplenic dysfunction.
- Chronic liver disease.
- Severe neuromuscular diseases.
Immunosuppression (including primary immunodeficiencies and those caused by HIV infection, drugs, including treatment with eculizumab, or in transplant recipients) and complement deficiencies.
- Cancer and malignant blood disorders.
Cochlear implant or awaiting an implant.
- Cerebrospinal fluid fistula.
- Celiac disease.
- Chronic inflammatory disease.
- Disorders and diseases that involve cognitive dysfunction: Down syndrome, dementia, etc.
- People institutionalized for an extended period.
- Pregnant women.

**Table A3:** Vaccination of cases diagnosed with influenza in primary care.

Vaccination				
	Total Cases	Yes	No	*p*-Value
	n (%)	n (%)	n (%)	
Total cases	6804	888	5916	
Sex				0.010
Male	3251 (47.8%)	388 (43.7%)	2863 (48.4%)	
Female	3553 (52.2%)	500 (56.3%)	3053 (51.6%)	
Age				<0.001
[0–2)	448 (6.58%)	68 (7.66%)	380 (6.42%)	0.190
[2–4)	800 (11.8%)	70 (7.88%)	730 (12.3%)	<0.001
[5–14)	1366 (20.1%)	54 (6.08%)	1312 (22.2%)	<0.001
[15–64)	3310 (48.6%)	239 (26.9%)	3071 (51.9%)	<0.001
[65 or over)	880 (12.9%)	457 (51.5%)	423 (7.15%)	<0.001
Severity				0.492
Mild	6717 (98.7%)	874 (98.4%)	5843 (98.8%)	
Complicated	87 (1.28%)	14 (1.58%)	73 (1.23%)	
Risk				<0.001
Yes	1580 (23.2%)	476 (53.6%)	1104 (18.7%)	
No	5224 (76.8%)	412 (46.4%)	4812 (81.3%)	
