# Supplementary material for: Association Between the Introduction of Pediatric Influenza Vaccination and Influenza Diagnoses in Primary Care and Hospitalizations: An Interrupted Time Series Study
Source: Vaccines (Basel). 2026 Apr 22;14(5):372. doi: 10.3390/vaccines14050372 (PMC13211677; doi:10.3390/vaccines14050372)
Supplement: Supplementary file 1 [file vaccines-14-00372-s001.zip › vaccines-4259452-supplementary.pdf]

SUPPLEMENTARY MATERIAL

Table S1. Adjusted negative binomial regressions for changes over time and by age groups. 0-2 years group and 2-4 years group. Vaccination period time: change in trend during the vaccination period. Vaccination start period: immediate effect of the vaccination period. Time since COVID onset: change in trend after the onset of COVID. COVID onset: immediate effect of the onset of COVID. Time: temporal trend.

	0-2 YEARS						2-4 YEARS					
	PRIMARY CARE			HOSPITAL			PRIMARY CARE			HOSPITAL		
	RR	CI 95%	P-value	RR	CI95%	P-value	RR	CI 95%	P-value	RR	CI 95%	P-value
**Time**	0,93	(0,79; 1,13)	1	0,97	(0,88; 1,09)	1	1,00	(0,79; 1,34)	1	0,97	(0,88; 1,07)	1
**COVID onset**	5,14	(0,04; 1173,7)	1	0,05	(0; 2,98)	0,29	2,34	(0,01; 730,12)	1	0,18	(0,01; 4,01)	0,957
**COVID Onset time**	1,06	(0,84; 1,33)	1	1,12	(0,95; 1,34)	0,39	1,00	(0,72; 1,31)	1	1,08	(0,95; 1,25)	0,711
**Start of vacci-nation period**	2,18	(0,21; 21,56)	1	0,25	(0,03; 2,07)	0,47	2,86	(0,2; 45,03)	1	0,39	(0,05; 2,6)	1
**Vacci-nation period**	0,97	(0,82; 1,18)	1	0,94	(0,79; 1,11)	1	0,95	(0,78; 1,17)	1	0,97	(0,83; 1,12)	1

Table S2. Adjusted negative binomial regressions for changes over time and by age groups. 5-14 years group and 15-64 years group. Vaccination period time: change in trend during the vaccination period. Vaccination start period: immediate effect of the vaccination period. Time since COVID onset: change in trend after the onset of COVID. COVID onset: immediate effect of the onset of COVID. Time: temporal trend.

	5-14 YEARS						15-64 YEARS					
	PRIMARY CARE			HOSPITAL			PRIMARY CARE			HOSPITAL		
	RR	CI 95%	P-value	RR	CI 95%	P-value	RR	CI 95%	P-value	RR	CI 95%	P-value
**Time**	1,03	(0,8; 1,41)	1	0,96	(0,81; 1,11)	1	1,01	(0,89; 1,16)	1	1,03	(0,89; 1,21)	1
**COVID onset**	1,69	(0,01; 568,97)	1	0,03	(0; 1,61)	0,175	0,07	(0,01; 0,92)	0,007	0,04	(0; 0,73)	0,004
**COVID Onset time**	1,02	(0,72; 1,38)	1	1,19	(0,99; 1,46)	0,115	1,09	(0,94; 1,28)	0,701	1,05	(0,88; 1,24)	1
**Start of vacci-nation period**	1,05	(0,04; 25,95)	1	0,25	(0,04; 1,44)	0,258	1,03	(0,18; 6,91)	1	0,46	(0,06; 3,94)	1
**Vacci-nation period**	0,91	(0,73; 1,13)	0,96	0,88	(0,76; 1,02)	0,146	0,87	(0,78; 0,99)	**0,011**	0,91	(0,79; 1,05)	0,347

Table S3. Adjusted negative binomial regressions for changes over time and by age groups. > 65 years group. Vaccination period time: change in trend during the vaccination period. Vaccination start period: im- 374 mediate effect of the vaccination period. Time since COVID onset: change in trend after the onset of COVID. COVID onset: immediate effect of the onset of COVID. Time: temporal trend.

	65 O MÉS					
	PRIMARY CARE			HOSPITAL		
	RR	CI 95%	P-value	RR	CI 95%	P-value
**Time**	0,94	(0,78; 1,13)	1	0,94	(0,78; 1,13)	1
**COVID onset**	0,97	(0,05; 40,85)	1	0,97	(0,05; 40,85)	1
**COVID Onset time**	1,09	(0,89; 1,35)	1	1,09	(0,89; 1,35)	1
**Start of vacci-nation period**	2,19	(0,35; 16,82)	1	2,19	(0,35; 16,82)	1
**Vacci-nation period**	0,95	(0,83; 1,1)	1	0,95	(0,83; 1,1)	1

Table S4. Adjusted negative binomial regressions for changes over time and by age groups after applying a sensitivity test. (The 2020/21 season has been excluded). 0-2 years group and 2-4 years group. Vaccination period time: change in trend during the vaccination period. Vaccination start period: im- 374 mediate effect of the vaccination period. Time since COVID onset: change in trend after the onset of COVID. COVID onset: immediate effect of the onset of COVID. Time: temporal trend.

	0-2 YEARS						2-4 YEARS					
	PRIMARY CARE			HOSPITAL			PRIMARY CARE			HOSPITAL		
	RR	CI 95%	P-value	RR	CI 95%	P-value	RR	CI 95%	P-value	RR	CI 95%	P-value
**Time**	0,94	(0,79; 1,13)	1	0,98	(0,88; 1,09)	1	1,00	(0,8; 1,33)	1	0,97	(0,88; 1,07)	1
**COVID onset**	5,14	(0,04; 1173,7)	1	0,05	(0; 2,98)	0,297	7,64	(0,03; 4192,16)	1	0,18	(0,01; 4,01)	0,957

**COVID Onset time**	1,06	(0,84; 1,33)	1	1,12	(0,95; 1,34)	0,397	0,96	(0,7; 1,27)	1	1,08	(0,95; 1,25)	0,711
**Start of vacci-nation period**	2,18	(0,21; 21,56)	1	0,25	(0,03; 2,07)	0,47	4,10	(0,3; 62,39)	0,62	0,39	(0,05; 2,6)	1
**Vacci-nation period**	0,98	(0,82; 1,18)	1	0,94	(0,79; 1,11)	1	0,99	(0,8; 1,22)	1	0,97	(0,83; 1,12)	1

Table S5. Adjusted negative binomial regressions for changes over time and by age groups after applying a sensitivity test. (The 2020/21 season has been excluded). 5-14 years group and 15-64 years group. Vaccination period time: change in trend during the vaccination period. Vaccination start period: immediate effect of the vaccination period. Time since COVID onset: change in trend after the onset of COVID. COVID onset: immediate effect of the onset of COVID. Time: temporal trend.

	**5-14 YEARS**						**15-64 YEARS**					
	**PRIMARY CARE**			**HOSPITAL**			**PRIMARY CARE**			**HOSPITAL**		
	**RR**	**IC 95%**	**P-value**	**RR**	**IC 95%**	**P-value**	**RR**	**CI 95%**	**P-value**	**RR**	**CI 95%**	**P-value**
**Time**	1,03	(0,8; 1,41)	1	0,96	(0,81; 1,11)	1	1,01	(0,89; 1,16)	1	1,03	(0,89; 1,22)	1
**COVID onset**	3,46	(0,01; 1585,77)	1	0,03	(0; 1,61)	0,175	0,13	(0,01; 2,78)	0,129	0,04	(0; 1,09)	0,013
**COVID Onset time**	0,99	(0,7; 1,35)	1	1,19	(0,99; 1,46)	0,115	1,08	(0,92; 1,26)	1	1,05	(0,87; 1,24)	1
**Start of vacci-nation period**	1,33	(0,06; 32,09)	1	0,25	(0,04; 1,44)	0,258	1,24	(0,21; 8,56)	1	0,47	(0,06; 4,24)	1
**Vacci-nation period**	0,93	(0,74; 1,16)	1	0,88	(0,76; 1,02)	0,146	0,89	(0,79; 1,02)	0,055	0,91	(0,79; 1,05)	0,446

Table S6. Adjusted negative binomial regressions for changes over time and by age groups after applying a sensitivity test. (The 2020/21 season has been excluded). > 65 years group. Vaccination period time: change in trend during the vaccination period. Vaccination start period: im- 374 mediate effect of the vaccination period. Time since COVID onset: change in trend after the onset of COVID. COVID onset: immediate effect of the onset of COVID. Time: temporal trend.

	**65 OR OVER**					
	**PRIMARY CARE**			**HOSPITAL**		
	**RR**	**CI 95%**	**P-value**	**RR**	**CI 95%**	**P-value**
**Time**	0,94	(0,78; 1,13)	1	0,94	(0,78; 1,13)	1
**COVID onset**	0,97	(0,05; 40,85)	1	0,97	(0,05; 40,85)	1
**COVID Onset time**	1,09	(0,89; 1,35)	1	1,09	(0,89; 1,35)	1
**Start of vaccination period**	2,19	(0,35; 16,82)	1	2,19	(0,35; 16,82)	1
**Vaccination period**	0,96	(0,83; 1,1)	1	0,96	(0,83; 1,1)	1
